# Neuroprotective Effects of Metformin and Berberine in Lipopolysaccharide-Induced Sickness-Like Behaviour in Mice

**DOI:** 10.1155/2024/8599268

**Published:** 2024-09-21

**Authors:** Triveni Kodi, Sharanya Praveen, Sravan Kumar Paka, Runali Sankhe, Adarsh Gopinathan, Nandakumar Krishnadas, Anoop Kishore

**Affiliations:** Department of Pharmacology Manipal College of Pharmaceutical Sciences Manipal Academy of Higher Education, Manipal 576104, Karnataka, India

## Abstract

Sickness behaviour, a set of behavioural changes associated with neuroinflammation, is expressed as decreased mobility and depressed behaviour. Activation of AMP-activated protein kinase (AMPK) is reported to regulate inflammation in conditions such as Alzheimer and traumatic brain injury. Metformin, an antidiabetic agent acting via AMPK activation, possesses anti-inflammatory properties. Similarly, the reported anti-inflammatory activities of berberine could be partially attributed to its ability to activate AMPK. In this study, we investigated the effects of metformin and berberine against lipopolysaccharide (LPS)-induced sickness-like behaviour, associated with neuroinflammation, impaired cognition, and oxidative stress. Swiss albino mice were divided into four groups, normal control, LPS control, metformin treatment, and berberine treatment. The control groups received saline for 7 days. Groups 3 and 4 received metformin (200 mg/kg) and berberine (100 mg/kg), respectively, orally once daily for 7 days. On day 7, 1 h after the treatments, animals received LPS (1.5 mg/kg i.p.) to induce sickness-like behaviour. Open field test (OFT) and forced swim test (FST), were performed within 2 h of LPS administration. Then, proinflammatory cytokines (IL-1*β* and TNF-*α*), acetylcholinesterase activity (AChE), and oxidative stress markers were estimated in the brain homogenate. In the LPS control group, immobility state, proinflammatory cytokines, AChE, and lipid peroxidation were significantly increased, whereas the glutathione levels were decreased. Pretreatment with metformin significantly improved immobility in the FST, with reduced IL-1*β*, oxidative stress markers, and AChE activity. However, no significant changes were observed in OFT. Berberine pretreatment exhibited only an apparent, statistically insignificant, improvement in sickness-like behaviour assessed using FST and OFT, cytokine levels, oxidative markers, and AChE. Several factors affect treatment efficacy, such as treatment duration and administered dose. Considering these, berberine warrants elaborate preclinical evaluation for neuroinflammation. Nevertheless, based on the effects observed, AMPK activators could regulate neuroinflammation, cognition, and oxidative stress linked with sickness-like behaviour.

## 1. Introduction

Inflammation plays an important role in the aetiology of depression [1]. Neuroinflammation with elevated levels of cytokines is linked to depression-like symptoms in humans [2, 3]. In rodents, intraperitoneal administration of lipopolysaccharide (LPS) triggers certain immune reactions that cause acute sickness-like behaviour. The sickness-like behaviour, which resembles a depressive-like state, is exhibited as decreased movement, tiredness, cognitive decline, and an inability to enjoy otherwise normally pleasurable situations [4, 5] Sickness-like behaviour causes immobility during forced swim test (FST) and tail suspension test (TST) and decreases locomotor activity in the open field test (OFT) [6–9].

The exact mechanism by which peripherally administered LPS affects the central nervous system (CNS) is not known. However, reports indicate that LPS binds to toll-like receptors and activates the NF-*κ*B pathway resulting in the release of proinflammatory cytokines [10, 11] The peripherally produced cytokines cross the blood-brain barrier (BBB) to enter the CNS, producing the neuroinflammation [12, 13]. The central effects of the cytokines involve the induction of sickness-like behaviour, a cluster of symptoms, which are generally observed in neurological disorders such as depression [14].

AMPK has been identified as an essential regulator in neuroinflammation, which activates anti-inflammatory systems [15–17]. In rodent models of LPS-induced memory deficit, the arrest of inflammation by suppression of NF-*κ*B release and improvement in memory deficits by metformin has been proven to be mediated via AMPK [18–20]. Currently, AMPK activators are being evaluated as drug candidates for conditions involving neuroinflammation.

Metformin, a clinically used antidiabetic drug that acts by activating AMP-activated protein kinase (AMPK) [21, 22] has shown promising effects against cognitive deficits [23, 24], stroke [25], arthritis [26], depression [27], inflammation [28, 29], etc., in preclinical models. Berberine is a naturally occurring isoquinoline alkaloid and a reported AMPK activator. In preclinical studies, it has shown several beneficial effects such as antidepressant [30, 31], anti-inflammatory [32, 33] antioxidant [34–37], antiamnesic [38, 39], antipsychotic, and anxiolytic [40, 41]. Berberine suppressed arachidonic acid and cyclooxygenase-2 expression via arresting NF-*κ*B signalling [42–44]. Activation of AMPK and Nrf2 by berberine inhibited proinflammatory cytokines and reduced oxidative stress [34, 45].

In the current study, we have explored the effect of the two AMPK activators, metformin and berberine, on their efficacy in improving the sickness-like behaviour induced by LPS administration, and the associated neuroinflammation. Metformin was selected as it is a well-known and established AMPK activator, serving as the standard reference drug. Berberine, reported to possess AMPK activating properties, was chosen as the test molecule to assess its effects on the model of neuroinflammation. Observations from this study could reveal whether metformin and berberine have any effects in alleviating LPS-induced sickness-like behaviour, thereby expanding their therapeutic applicability in neuroinflammatory disorders.

## 2. Materials and Methods

### 2.1. Experimental Animals

Sixteen male Swiss albino mice (8–10 weeks old) weighing 20−30 g were obtained from the Central Animal Research Facility (CARF), Manipal Academy of Higher Education, Manipal, India. All the animals were housed in sterile polypropylene cages, at a temperature of 23±2°C and humidity of 50 ± 5% with 12 h light/dark cycle. The animals were fed a standard rodent diet in the pellet form and provided water *ad libitum*. The study was approved by the Institutional Animal Ethics Committee (IAEC) (no. IAEC/KMC/114/2021), Kasturba Medical College, Manipal Academy of Higher Education, Manipal, India. The animal experiments were carried out as per the guidelines for the use and care of laboratory animals by the CCSEA (Committee for Control and Supervision of Experiments on Animals), Government of India.

### 2.2. Drugs and Chemicals

Metformin and berberine were obtained from TCI Chemicals, India. LPS from *Escherichia coli* 0111: B4 was purchased from Sigma-Aldrich, St. Louis, MO, USA. Ellman's reagent, sulfanilamide, and trichloroacetic acid were procured from Sisco Research Laboratories, India. The bicinchoninic acid (BCA) protein assay kit was procured from Cyanagen (QPRO), Italy, and the remaining chemicals were from Sisco Research Laboratories.

### 2.3. Preparation and Administration of Drugs

LPS was prepared as a solution in sterile, endotoxin-free cold 0.9% NaCl solution, and injected intraperitoneally at a dose of 1.5 mg/kg to induce sickness-like behaviour and neuroinflammation. The dose of LPS (1.5 mg/kg) was selected based on the previous experiments carried out in our lab [6, 46]. The test compounds, metformin and berberine, were prepared as solutions in normal saline and administered orally. The dose of metformin 200 mg/kg [16, 46] and berberine 100 mg/kg [18] were selected based on published reports.

### 2.4. Experiments and Behavioural Tests

**Figure 1:**
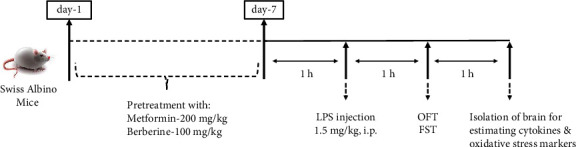
Schematic representation of the experimental protocol.

The study involved pretreating two groups of mice (namely, groups 3 and 4) with the test drugs, orally, for 7 days. Group 3 was administered metformin in a dose of 200 mg/kg of body weight, and group 4 was given berberine in a dose of 100 mg/kg of body weight. On day 7, one hour after the drug administration, a single dose of LPS solution (1.5 mg/kg, b.w.) was injected intraperitoneally to induce sickness-like behaviour. One hour later, an open field test (OFT) and forced swim test (FST) were performed on all the animals. Three hours post-LPS administration, the animals were euthanized, and whole brain samples were collected. The brains were homogenized in ice-cold 0.1 M phosphate buffer (pH 7.4), and the supernatant was collected for the estimation of catalase, GSH, lipid peroxidation, acetylcholinesterase enzyme activity, and cytokine (IL-1beta and TNF-alpha) levels (Figure 1). Two additional groups of animals were included, normal control (group 1, administered saline orally for 7 days) and LPS control (group 2, administered saline orally for 7 days and LPS solution on day 7), for the comparison of efficacy (Table 1).

#### 2.4.1. Open Field Test

OFT was performed to determine the locomotor activity and exploratory behaviour of animals. Mice were individually placed in the centre square of the OFT glass chamber (30 cm × 30 cm × 60 cm), divided into 9 equal squares (10 cm × 10 cm each), observed for 6 min, and assessed for the number of line crossings, grooming, rearing, and centre square entries. After each trial, the chamber was cleaned with 70% ethanol to mask any odor-related signals [46].

#### 2.4.2. Forced Swim Test

FST was performed to assess the behavioural despair of animals. Mice were individually allowed to swim in a transparent cylinder tank (30 × 20 cm) and the immobility time spent by the mice was measured for 6 min [46].

### 2.5. Antioxidant Assays

#### 2.5.1. Estimation of Lipid Peroxidation

The amount of malondialdehyde (MDA) in the brain homogenate was quantified according to the procedure given by the authors in [47]. In brief, equal parts of brain homogenate and TBA-TCA-HCL reagent were mixed in a test tube and heated at 90°C for 30 min. MDA in the homogenate forms an adduct with two molecules of thiobarbituric acid (TBA) to produce a pink colour. Then, the tubes were centrifuged at 10000 rpm for 10 min at 4°C. 100 *μ*l of the supernatant was transferred to a 96-well plate. The amount of MDA formed was measured calorimetrically at 532 nm using a microplate reader and the values were expressed as nmol/mg of protein.

#### 2.5.2. Estimation of Reduced Glutathione (GSH)

The amount of GSH in the brain homogenate was determined according to the procedure described by the authors in [48]. In brief, equal parts of brain homogenate and 5% TCA reagent were taken in a test tube and centrifuged at 4000 rpm for 10 min at 4°C. The supernatant was collected and incubated at 37°C with phosphate-buffered saline (PBS) (pH = 8) and 5,5-dithiobis-(2-nitrobenzoic acid) (DTNB) solution for 10 min in a 96-well plate covered with aluminium foil. GSH reacts with DTNB to form 5′-thio-2-nitrobenzoic acid (TNB), a yellow-coloured compound. The GSH levels were measured calorimetrically at 412 nm using a microplate reader and the values were expressed as µmol/mg of protein.

#### 2.5.3. Estimation of Catalase

Catalase activity in the brain homogenate was estimated using the procedure described by the authors in [49]. Catalase converts hydrogen peroxide to water and oxygen. Catalase activity is determined by measuring its ability to decompose H_2_O_2_. Briefly, to 900 *µ*l of the brain homogenate was added to 900 *µ*l of phosphate-buffered saline hydrogen peroxide (PBS-H2O2) solution taken in a cuvette, 15 µl of brain homogenate was added and mixed well. The absorbance (at 240 nm) of the mixture was measured for 60 s, using a UV-visible spectrophottometer (kinetic method). A decrease in absorbance over 60 s is directly proportional to the catalase activity present in the tissue sample. The catalase activity was expressed as *µ*mol of H_2_O_2_ decomposition/minute/mg of protein.

#### 2.5.4. Estimation of Acetylcholinesterase (AChE) Enzyme Activity

The acetylcholinesterase enzyme activity in the supernatant of brain homogenate was determined using the method reported by the authors in [50]. 100 *μ*l of the supernatant was added to a cuvette containing 650 *µ*l of 0.1 phosphate buffer (pH 8). To this mixture, 5 *μ*l of acetylthiocholine iodide was added, followed by 25 *μ*l of 5,5-dithiobis-(2-nitrobenzoic acid) (DTNB), to start the reaction. Acetylcholinesterase breaks down acetylthiocholine iodide into acetate and thiocholine. Thiocholine forms a yellow-coloured adduct upon interaction with DTNB. The amount of thiocholine produced and the intensity of the yellow-coloured adduct are directly proportional to AChE activity. The absorbance was measured for 4 min at 412 nm using a UV-visible spectrophotometer and per-minute change in absorbance was calculated. The enzyme activity is expressed in *μ*moles of acetylthiocholine iodide hydrolyzed/minute/mg of protein.

#### 2.5.5. Estimation of Brain Cytokines Levels

Brain IL-1*β* and TNF-*α* were estimated using Mouse IL-1*β* and TNF-*α* enzyme-linked immunosorbent assay ELISA kits (GENLISA™, Krishgen Biosystems, India), respectively. The assays were carried out following the manufacturer's instructions. The results were interpolated from the standard curve constructed based on the provided IL-1*β* and TNF-*α* standards.

#### 2.5.6. Statistical Analysis

The data were analysed using the software GraphPad Prism 8.4 (GraphPad Software Inc., San Diego, CA, USA). All results were expressed as mean ± S.E.M. The mean of each group was compared with the means of every other group using one-way analysis of variance (ANOVA) followed by Tukey's post hoc test. A *p* value of <0.05 was considered significant.

## 3. Results

### 3.1. Effect of Metformin and Berberine on LPS-Induced Sickness-Like Behaviour

**Figure 2:**
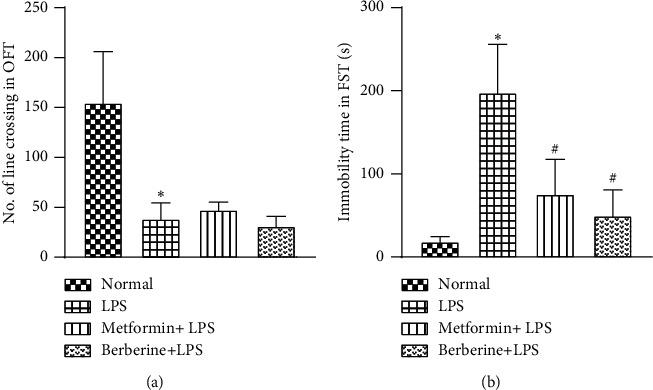
Effect of metformin and berberine on LPS-induced changes in locomotor activity and immobility: (a) number of line crossings in OFT and (b) immobility time (s) in FST. Statistical analysis was performed by one-way ANOVA followed by Tukey's multiple comparison tests. ^∗^*p* < 0.05 as compared to the normal group. ^#^*p* < 0.05 as compared to the LPS group.

A single dose of LPS (1.5 mg/kg, b.w.) significantly decreased the locomotor activity in mice (LPS control group), as seen by the decreased line crossings in OFT (38.25 ± 8 in the LPS control group vs 154.5 ± 25.8 in the normal control group, at *p* < 0.05) (Figure 2(a)). Pretreatment with metformin and berberine did not improve the decreased locomotor activity induced by LPS administration (47.25 ± 4 and 31 ± 5, respectively, *F* [3, 12] = 17.52, *R*^2^ = 0.8141).

In FST, LPS-challenged mice exhibited significant immobility (197.2 ± 29.2 s as compared to 18.1 ± 3 s of the normal control group at *p* < 0.05) (Figure 2(b)). Pretreatment with metformin and berberine significantly improved LPS-induced immobility (75.2 ± 21 and 49.3 ± 15.6, respectively, *p* < 0.05, *F* [3, 12] = 15.82, *R*^2^ = 0.7982).

### 3.2. Effect of Metformin and Berberine on Oxidative Stress Markers in the Brain

#### 3.2.1. Lipid Peroxidation

**Figure 3:**
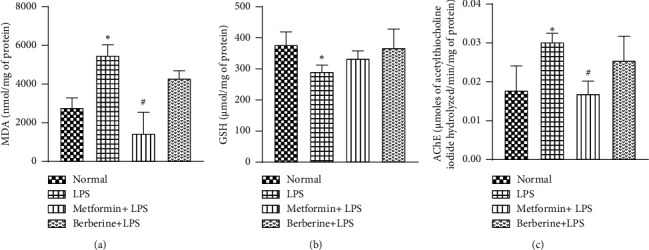
Effect of metformin and berberine against LPS-induced changes in (a) MDA levels (nmol/mg of protein), (b) GSH levels (*µ*mol/mg of protein), and (c) AChE levels (*μ*moles of acetylthiocholine iodide hydrolyzed/min/mg of protein). ^∗^*p* < 0.05 as compared to the normal group. ^#^*p* < 0.05 as compared to the LPS group.

There was a significant increase in lipid peroxidation (as observed by elevated MDA levels (nmol/mg of protein)) in the brain homogenate supernatant in the LPS control group as compared to the normal animals (5479.2 ± 275.8 vs 2776.9 ± 252, respectively, *p* < 0.05) (Figure 3(a)). Animals pretreated with metformin showed a significant reduction in the MDA levels when compared with the LPS control group (14441.9 ± 551.3, *p* < 0.05, *F* [3, 12] = 25.83, *R*^2^ = 0.8659). However, there was no significant reduction in the MDA levels in the berberine-pretreated group (4304 ± 193.8).

#### 3.2.2. Reduced Glutathione Levels

There was a significant decrease in GSH levels (µmol per mg of protein) in LPS-challenged animals as compared to the normal group (291.6 ± 10.2 vs 377.7 ± 20.7, *p* < 0.05) (Figure 3(b)). There was an apparent, but statistically insignificant, improvement in the GSH levels with metformin and berberine pretreatment (334.9 ± 11.9, 367.9 ± 30.1, respectively, *F* [3, 12] = 3.811, *R*^2^ = 0.4879).

#### 3.2.3. Effect of Metformin and Berberine on AChE Enzyme Activity in the Brain

LPS-challenged animals showed a significant increase in AChE activity (*μ*moles of acetylthiocholine iodide hydrolyzed/min/mg of protein) as compared to the normal group (0.03 ± 0.001 vs 0.01 ± 0.003, *p* < 0.05) (Figure 3(c)). A significant reduction in the AChE activity was observed in the metformin pretreated animals (0.01 ± 0.001, *p* < 0.05, *F* [3, 12] = 6.88, *R*^2^ = 0.6324) as compared to LPS-challenged animals. However, no significant reduction was observed in the berberine pretreated group (0.02 ± 0.003).

### 3.3. Effect of Metformin and Berberine on IL-1*β* and TNF-*α* Cytokine Levels in the Brain

**Figure 4:**
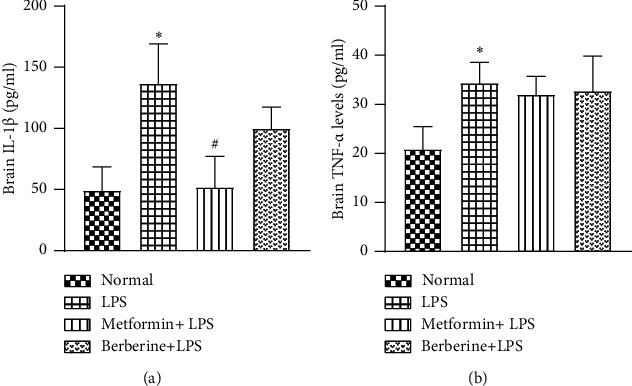
Effect of metformin and berberine on IL-1*β* and TNF-*α* cytokine levels in the brain homogenates: (a) IL-1*β* (pg/ml) and (b) TNF-*α* levels (pg/ml). ^∗^*p* < 0.05 as compared to the normal group. ^#^*p* < 0.05 as compared to the LPS group.

LPS-challenged animals exhibited a significant increase in IL-1*β* levels (pg/ml) as compared to the normal group (136.7 ± 18.8 vs 49.4 ± 11.1, *p* < 0.05) (Figure 4(a)). In the metformin pretreated group, there was a significant reduction in the IL-1*β* levels (52.1 ± 14.3, *p* < 0.05, *F* [3, 8] = 8.922, *R*^2^ = 0.7699) as compared to LPS-challenged animals. There was no reduction in the IL-1*β* levels with berberine pretreatment (100.2 ± 10.02).

There was a significant elevation in the TNF-*α* levels (pg/ml) in LPS-challenged animals as compared to the normal group (34.41 ± 2.3 vs 20.9 ± 2.6, *p* < 0.05) (Figure 4(b)). However, metformin and berberine pretreated groups did not show any reduction in the TNF-*α* levels (32 ± 2 and 32.7 ± 4, respectively, *F* [3, 8] = 4.477, *R*^2^ = 0.6267).

## 4. Discussion

In rodents, the peripheral administration of the bacterial endotoxin, LPS, initially activates the immune system in the periphery which eventually triggers neuroinflammation in the CNS [51]. This results in sickness-like behaviour. The sickness-like behaviour appears within 3-4 h of LPS administration and is characterized by severe immobility, fever, anhedonia, hyperalgesia, lack of exploratory behaviour, and social interaction [52–55]. The LPS model is widely employed to investigate inflammation-related sickness-like behaviour in rodents [1]. In the current study, we have assessed the effect of metformin and berberine against the LPS-induced sickness-like behaviour in mice.

LPS binds to toll-like receptors (TLRs), induces phosphorylation of *inhibitory protein κB* (I*κ*B), and causes translocation of *nuclear factor-κB* (NF-*κ*B) into the nucleus. This induces the expression of several cytokines, chemokines, and growth factors that trigger inflammatory responses [56–58]. The rise in the levels of the cytokines in turn enhances NF-*κ*B signalling, thus triggering a vicious cycle [59, 60]. These cytokines, such as TNF-*α* and IL-1*β*, reach the brain and induce neuroinflammation that is exhibited as sickness-like behaviour [4, 12, 61]. These proinflammatory cytokines (TNF-*α* and IL-1*β*) are reported to play an important role in the induction of sickness-like behaviour [62]. Therefore, in the current study, the levels of TNF-*α* and IL-1*β* in the whole brain homogenate were used to assess the extent of inflammatory activation.

NF-*κ* B is also activated by LPS-induced phosphorylation of mitogen-activated protein kinases (MAPKs). This initiates a cascade of reactive oxygen pathways and nitrosative stress in cells [57, 63–65]. Oxidative stress enhances proinflammatory cytokines and neuroinflammation [66], and the reduction in oxidative stress is reported to ameliorate neuroinflammation [67, 68]. The levels of oxidative markers such as lipid peroxidation, catalase, and GSH were estimated in the study as markers of inflammation and to ascertain the efficacy of the test drugs.

In this preclinical model, the neuroinflammation triggered in the CNS by peripheral LPS administration is a generalized response, spanning multiple regions of the brain [69]. Also, since this is an acute model where the behavioural studies were conducted within 2 hours, and the animals were sacrificed, and brains isolated within 3 hours of LPS administration, the effects of the toxicant on memory, locomotor activity, alertness, etc., are short-term effects. Therefore, the estimation of biochemicals (inflammatory and oxidative markers) on the whole brain homogenate was considered more relevant than estimating their levels in specific brain areas such as the hippocampus and frontal cortex.

Berberine is reported to exhibit a neuroprotective effect against inflammation via the modulation of SIRT1/p38 MAPK signalling and NLRP3 inflammasome [31, 70, 71]. It repressed proinflammatory responses by mitigating MAPK activation via enhancing AMPK activation in macrophages [34, 45] and exhibited antidepressant-like effects [72]. It mediated the activation of AMPK and p38, promoted Nrf2, and suppressed proinflammatory cytokines and oxidative stress [34, 73]. Therefore, we evaluated the effect of berberine on LPS-induced sickness-like behaviour, a condition with overlapping symptoms as that of clinical depression. To compare the effects of berberine, we have used metformin, the clinically used AMPK activator, as the standard.

In mice, the forced swim test and open field test are among the behavioural tests employed to quantify the sickness-like behaviour induced by LPS. The immobility time in FST and the number of line crossings in OFT are reduced after LPS administration [74, 75]. We have observed that pretreatment with metformin and berberine mitigated LPS-induced severe immobility in the forced swim test in mice. However, they failed to exhibit any significant improvement in the number of line crossings in OFT. Such a discrepancy, where a molecule shows improved activity in FST but not in OFT has also been reported previously using similar models [30, 72, 76–78]. We assume that the reason for such an effect could lie in the fundamental differences in the aetiologies of the two conditions. Mechanistic explorations along these lines are needed to understand these differences.

LPS challenge significantly increased the brain levels of IL-1*β* and TNF-*α*, suggesting a rise in the LPS-induced proinflammatory cytokine responses in the brain. These results are consistent with the previous studies where IL-1*β* and TNF-*α* levels were enhanced in the brain after peripheral treatment of LPS [77, 79]. Here, we have observed that metformin alleviated the levels of IL-1*β* [80] without any apparent effect on TNF-*α* levels. There are multiple reports where metformin has exhibited a decrease in TNF-*α* levels. However, there are also *in vitro* research works which suggest that metformin may not have any significant effect on the TNF-*α* levels elevated by LPS administration [81]. The reason for such contradicting results is not known.

Pretreatment with berberine also exhibited a similar response, where the elevated IL-1*β* levels in the whole brain homogenate were ameliorated without any apparent effect on TNF-*α* levels. There are several reports on berberine where the effect of the drug on cytokine levels has been inconsistent. Berberine has ameliorated LPS-induced inflammation and apoptosis, mediated by the NF-*κ*B signalling in *β*-cells [82], human monocytic cells [83], and in obese mice induced by a high-fat diet [84]. However, it enhanced IL-1*β* levels upon microglial activation [85]. In addition, berberine failed to show any effect on the IL-1*β* levels in metabolic syndrome and related disorders [71, 86].

Consistent with the previous reports, peripheral administration of LPS enhanced oxidative stress [87], evident as increased lipid peroxidation [71, 88] and decreased GSH levels [71, 89]. In the present study, pretreatment with metformin significantly attenuated enhanced MDA levels. These findings were corroborated by other researchers [90, 91]. However, the GSH levels were not significantly enhanced by the metformin pretreatment in our study. Pretreatment with berberine did not significantly attenuate MDA levels or restore GSH levels. These results are consistent with [86], where berberine did not show significant changes in oxidative stress biomarkers. We presume that these insignificant findings could be dependent on berberine's dose, route of administration, time, as well as duration of administration. LPS administration significantly upregulates the AChE activity in the CNS, indicating a reduction in cholinergic activity [92–94]. In our study, LPS significantly elevated the AChE activity, and treatment with metformin attenuated its levels. Similar results with metformin have been obtained earlier [95]. However, berberine did not exhibit any reduction in AChE activity, which is consistent with previous reports [71].

This current study has certain limitations. Even though intraperitoneal LPS injection in mice is a widely used model to screen drugs against sickness-like behaviour, the exact mechanism by which LPS induces this effect is not known. Though the involvement of inflammatory mediators in this phenomenon is evident, the details of the interplay between these mediators and the CNS neurotransmitters are also not known. In addition, in this preclinical model, the impairment in memory and alterations in behaviour are the results of an acute inflammatory response (i.e., within 3 h of induction). Clinically, impairments in memory, depression, locomotion, etc., are seldom manifested in such a fashion. Therefore, studies involving chronic models are more useful for mechanistic assessment, where the effects of drugs on synaptic plasticity, expression and levels of neurotransmitters, and structural changes in the CNS can be assessed. Acute models are useful for preliminary screening and proof of concept studies. Also, we have used only a single dose of the test molecule, berberine (100 mg/kg, b.w.), in the study. At this dose, the molecule has shown only a partially beneficial response. A study involving higher doses of berberine and prolonging its duration of administration would have provided better insights into its efficacy. In addition, in-silico drug discovery approaches such as network pharmacology could be employed to identify possible targets of the molecules in inflammatory pathways and ligand-biomolecule docking studies could be performed to predict their activity biological activity [96].

In conclusion, metformin exhibits a significant beneficial effect against LPS-induced sickness-like behaviour in mice, whereas berberine shows only a partial response. Further mechanistic studies are warranted using higher doses of berberine to confirm its role in attenuating neuroinflammation. Nevertheless, our preliminary conclusion is that AMPK activators such as metformin and berberine could have a role in regulating neuroinflammation.

## Figures and Tables

**Table 1:** The treatment groups, drugs, and their doses.

Groups	Treatment	Drugs and the dose administered
Group 1	Normal control	Saline, administered orally (5 ml/kg, b.w.) for 7 days
Group 2	LPS control	LPS, administered intraperitoneally in a dose of 1.5 mg/kg of body weight on day 7
Group 3	Metformin + LPS	Metformin, administered orally in a dose of 200 mg/kg of body weight, orally for 7 days followed by LPS (1.5 mg/kg, i.p.) on day 7
Group 4	Berberine + LPS	Berberine, administered orally in a dose of 100 mg/kg of body weight, for 7 days followed by LPS (1.5 mg/kg i.p.) on day 7

## Data Availability

The data used to support the findings of this study are available from the corresponding author upon request.
